# Universal Strategy for Improving Perovskite Photodiode Performance: Interfacial Built‐In Electric Field Manipulated by Unintentional Doping

**DOI:** 10.1002/advs.202101729

**Published:** 2021-07-15

**Authors:** Dan Wu, Wenhui Li, Haochen Liu, Xiangtian Xiao, Kanming Shi, Haodong Tang, Chengwei Shan, Kai Wang, Xiao Wei Sun, Aung Ko Ko Kyaw

**Affiliations:** ^1^ Guangdong University Key Laboratory for Advanced Quantum Dot Displays Shenzhen Key Laboratory for Advanced Quantum Dot Displays and Lighting Department of Electrical & Electronic Engineering Southern University of Science and Technology Xueyuan Blvd. 1088 Shenzhen 518055 P. R. China; ^2^ College of New Materials and New Energies Shenzhen Technology University Lantian Road 3002 Shenzhen 518118 P. R. China; ^3^ Light, Nanomaterials, Nanotechnologies (L2n) Laboratory CNRS ERL 7004 and Department of Optical Nanotechnologies University of Technology of Troyes Troyes 10004 France

**Keywords:** interfacial built‐in electric field, perovskite photodiodes, unintentional doping

## Abstract

Organic–inorganic halide perovskites have demonstrated significant light detection potential, with a performance comparable to that of commercially available photodetectors. In this study, a general design guideline, which is applicable to both inverted and regular structures, is proposed for high‐performance perovskite photodiodes through an interfacial built‐in electric field (*E*) for efficient carrier separation and transport. The interfacial *E* generated at the interface between the active and charge transport layers far from the incident light is critical for effective charge carrier collection. The interfacial *E* can be modulated by unintentional doping of the perovskite, whose doping type and density can be easily controlled by the post‐annealing time and temperature. Employing the proposed design guideline, the inverted and regular perovskite photodiodes exhibit the external quantum efficiency of 83.51% and 76.5% and responsivities of 0.37 and 0.34 A W^−1^, respectively. In the self‐powered mode, the dark currents reach 7.95 × 10^−11^ and 1.47 × 10^−8^ A cm^−2^, providing high detectivities of 7.34 × 10^13^ and 4.96 × 10^12^ Jones, for inverted and regular structures, respectively, and a long‐term stability of at least 1600 h. This optimization strategy is compatible with existing materials and device structures and hence leads to substantial potential applications in perovskite‐based optoelectronic devices.

## Graphical abstract

A general design guideline, which is applicable to both inverted and regular structures, is proposed for high‐performance perovskite photodiodes through an interfacial built‐in electric field (*E*). The interfacial *E* can be modulated by unintentional doping of the perovskite, whose doping type and density can be easily controlled by the post‐annealing time and temperature.

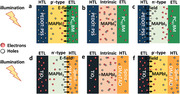

## 1 Introduction

Organic–inorganic halide perovskites have attracted considerable research attention in the past decade owing to their superior optoelectrical properties. The facile solution processability and semiconducting properties of perovskites have enabled researchers to fabricate many types of optoelectronic devices such as solar cells, light‐emitting diodes (LEDs), light‐emitting field‐effect transistors, and semiconductor lasers.^[^ ^1^, ^2^, ^3^, ^4^, ^5^, ^6^ ^]^ Scientists have also demonstrated high‐sensitivity perovskite‐based photodetectors, exploiting the advantages of high charge carrier mobility, long diffusion length, low dark current, and sharp absorption edge of hybrid perovskites.^[^ ^4^, ^7^, ^8^, ^9^ ^]^ Broadband photodetectors are used to sense a broad spectrum of light, for example, X‐rays, ultraviolet (UV) light, and visible light, whereas narrowband photodetectors are used to detect a narrow range of light for remote control, biomedical sensing, etc.^[^ ^10^, ^11^, ^12^, ^13^, ^14^, ^15^ ^]^ Additional features, such as sustainable and independent operation and portability are also required for some specific applications, and therefore, it is desirable for the photodetector to work in a self‐powered mode.^[^ ^16^, ^17^ ^]^ Despite various types of photodetectors, the photodetector performance is continuously improved toward the common goal of high detectivity and fast response speed at the detection wavelengths in combination with other specific features according to the targeted applications.

Generally, perovskite‐based photodetectors can be constructed into two structural configurations with different working mechanisms: lateral structures for phototransistor/photoconductors and vertical structures for photodiodes.^[^ ^8^, ^18^, ^19^ ^]^ The latter is better in terms of response time because of the sandwiched structure with a short distance between two electrodes, creating a short travel distance and transit time for charge carriers, resulting in a fast response time. Furthermore, self‐powered photodetectors can be achieved when photodiodes work at zero bias, which is similar to solar cells operating under short‐circuit conditions.^[^ ^4^, ^7^ ^]^ Several strategies have been proposed to improve perovskite photodiode performance. Nanostructures, such as diffractive gratings, 2D nanodisks, and butterfly light‐trapping structures have been incorporated into perovskites to enhance carrier generation upon light incidence.^[^ ^20^, ^21^, ^22^ ^]^ Carrier transport and collection have also been improved through the selection of suitable materials for respective transport layers.^[^ ^23^, ^24^ ^]^ However, most existing strategies are applicable only to specific device structures or certain material combinations. The two most widely used photodiode structures are inverted and regular structures, and a key problem in designing the highly efficient perovskite photodiodes is the necessity of multiple‐parameters‐optimization to obtain different optimized conditions for different structures, which is complicated and time‐consuming. The solution is not simply a fixed set of materials and processed parameters for all device structure types. In most cases, one set of optimized process parameters for one device structure is unsuitable for other types. Therefore, it is highly desirable to develop a general design guideline that is applicable to all device structures to further enhance the perovskite photodiode performance.

To develop a strategy for both inverted and regular structures, it is necessary to trace back to the intrinsic nature of perovskites. In recent years, there have been reports investigating the unintentional doping of perovskite materials and the formation of the p–n homojunction for efficient charge carrier separation and transport.^[^ ^25^, ^26^, ^27^ ^]^ Perovskite, for example MAPbI_3_, can be either p‐ or n‐type depending on the synthesis conditions, such as the annealing temperature and time, as well as the precursor ratio.^[^ ^14^, ^27^, ^28^ ^]^ The resultant MAPbI_3_ films with Pb^2+^‐rich/MA^+^‐deficient/I^−^‐deficient precursors become n‐doped, while those with MA^+^‐rich/Pb^2+^‐deficient precursors turn to p‐doping, owing to the presence of defects induced by vacancies or interstitial elements. Based on first‐principle density‐functional theory calculations, intrinsic point defects of vacancies (*V*_MA_, *V*_Pb_, *V*_I_), interstitial (MA_i_, Pb_i_, I_i_), substitutions (MA_Pb_, Pb_MA_), and antisite substitutions (MA_I_, Pb_I_, I_MA_, I_Pb_) can be formed with different formation energy.^[^ ^29^ ^]^ All these point defects create a transition level and are located near the conduction band (CB) minimum (valence band [VB] maximum) or within the forbidden band of MAPbI_3_. Moreover, most of the point defects have low formation energy, indicating that the defects are primarily shallow‐level electron acceptors or donors. Electron acceptors (e.g., I_i_, MA_Pb_, and *V*_Pb_) with the lowest formation energy can increase the hole carrier density, thus leading to the formation of p‐type perovskite. Similarly, electron donors (e.g., MA_i_ and *V*_I_) with the lowest formation energy can increase the electron carrier density and make n‐type perovskite. Although some studies have focused on one side of the heterojunction and perovskite doping density calculation by Mott–Schottky analysis,^[^ ^30^, ^31^, ^32^ ^]^ as well as the Kelvin probe force microscopy technique,^[^ ^33^, ^34^ ^]^ there are few reports considering the heterojunction formed at both interfaces between perovskite and adjacent charge transport layers and the influence of the interfacial built‐in electric field (*E*) on overall device performance. Interestingly, despite the wide discussion of the defect density and its carrier mobility influence, the intrinsic nature of the perovskite owing to unintentional doping and associated defect density was seldom considered in connection with perovskite photodiode performance. In fact, the unintentionally doped n‐ or p‐type perovskite active layer and adjacent transport layers at both sides would form heterojunctions at the interfaces where interfacial *E* would be built. This interfacial *E* would, in turn, significantly influence carrier separation and transport properties. Therefore, manifesting the hidden mechanism behind the unintentional doping of perovskite would offer a universal strategy for the optimization of different device structures without undergoing painstaking trial and error.

In this study, we investigated the interfacial *E* by unintentional doping of the perovskite and its influence on photodiode performance. Experimental results and numerical simulation data revealed that interfacial *E* generated at the interface away from the light incidence side is critical for achieving optimum device performance regardless of the device structure. This interfacial *E* sweeps out the photogenerated carriers to the respective electrodes, and hence, is favorable for charge collection and transport as long as the defect density inside the perovskite film is low enough to prevent charge recombination. The interfacial *E* strength and direction could be modulated by varying the perovskite doping type and density, which can be easily realized by controlling the annealing temperature and time. This universal strategy based on interfacial *E* manipulation simply by the unintentional doping of perovskite is naturally compatible with existing materials and is applicable to all device structures.

## 2 Results and Discussion

**Figure 1:**
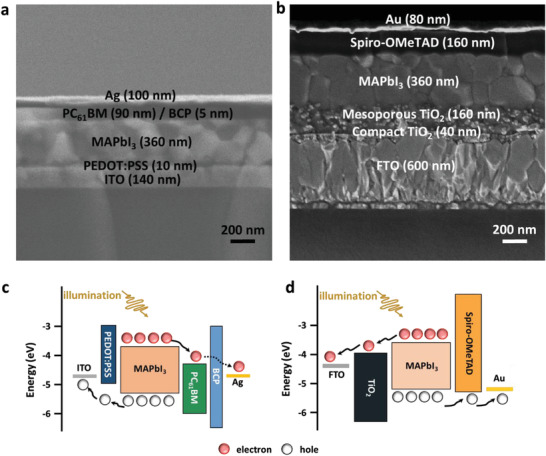
Cross‐sectional SEM images of both a) inverted and b) regular perovskite photodiodes and schematics of energy levels and the process of carrier generation, extraction, and transport of c) the inverted and d) regular perovskite photodiodes.

We investigated the inverted (indium tin oxide [ITO]/PEDOT:PSS/MAPbI_3_/PC_61_BM/BCP/Ag) and regular (fluorine‐doped tin oxide [FTO]/compact TiO_2_/mesoporousTiO_2_/MAPbI_3_/spiro‐OMeTAD/Au) structured self‐powered photodiodes. Cross‐sectional scanning electron microscope (SEM) images of both inverted and regular structures are shown in **Figure** **1**, where a smooth and uniform MAPbI_3_ can be clearly observed. The perovskite material as an active layer was sandwiched between the hole and electron transport layers (ETLs) in connection with the respective electrodes. The thickness of each layer is marked on the respective images.

Figure 1 shows the schematic energy level diagram and the process of carrier generation, separation, and transport. Upon absorption of incident photons by perovskites in both inverted and regular structures, photo‐induced carriers are generated. The electrons and holes drift to opposite directions. The electrons are injected into the ETL and transported to the cathode, whereas the holes are injected into the hole transport layer (HTL) and transported to the anode. The photoinduced electron and hole extraction occurs at the perovskite/ETL and perovskite/HTL interfaces, respectively. Without considering the perovskite doping type and density, the carrier extraction at the interfaces is mainly influenced by the energy levels of the respective functional layers. However, in a real device, light illuminates only one side of the device (transparent electrode side), and the photon‐induced carrier generation rate is the highest at the light‐incident side of the active layer. Therefore, one type of carriers (holes/electrons for the inverted/regular structure) will be instantly extracted to the transport layer (PEDOT:PSS/TiO_2_), whereas the other type of carriers (electrons/holes) must be transported across the entire active layer. If the extraction and transport of electrons/holes are not sufficiently efficient at the side far from the light incident side, the accumulated electrons/holes may form unwanted barriers for the extraction and transport of newly generated carriers, and nonradiative recombination may lower the overall efficiency. In contrast, if we specifically control the perovskite film doping type and density and create interfacial *E*_s_ at the perovskite/transport layer interfaces, the charge extraction can be rationally improved, especially at the far side from the light incident side, leading to a higher device performance. Hence, we designed heterojunctions at the interfaces between the perovskite and adjacent transport layers at both sides to generate interfacial *E*, according to regular or inverted device structures, to facilitate carrier extraction and transport, as shown in Figure 1.

To verify the influence of the perovskite doping type and density, and the influence of resultant *E* on the carrier extraction and transport, both inverted and regular structure perovskite photodiodes were simulated with FLUXiM Setfos 4.6.10, which is widely used to simulate various types of LEDs, and solar cells.^[^ ^35^, ^36^ ^]^ The simulated structures and respective thickness of each layer of the perovskite diodes are referred to in the cross‐sectional view in Figure 1. The simulation parameters are listed in Tables S1 and S2, Supporting Information.

**Figure 2:**
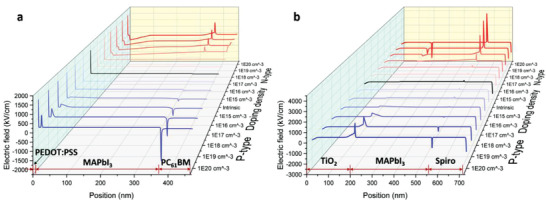
Simulated built‐in electric field for various perovskite doping type and densities. The position refers to the cross‐section of the a) inverted and b) regular structures.

At the interfaces between the active and transport layers, the interfacial *E*_s_ are constructed owing to energy level differences. There are two interfacial *E*_s_ located at each side of the active layer, as shown in **Figure** **2**. Upon changes in the perovskite doping type and density, the strength as well as the direction of the interfacial *E* change accordingly (see Figures S1 and S2, Supporting Information). For example, in the inverted structure photodiode, if the perovskite is unintentionally doped with n‐type (until the Fermi level lies above the lowest unoccupied molecular orbital (LUMO) of PC_61_BM) (Figure S1a,b, Supporting Information) because of the synthesis process, there is one *E* at the PEDOT:PSS/perovskite interface pointing from the perovskite to the PEDOT:PSS, while there is another small one at the perovskite/PC_61_BM interface, which is in the opposite direction. The *E* at the PEDOT:PSS/perovskite interface facilitates carrier transport to the respective electrodes, whereas the other inhibits carrier transport. When the perovskite is slightly n‐type doped (the Fermi level lies below the LUMO of PC_61_BM) (Figure S1c, Supporting Information), the *E* at the PEDOT:PSS/perovskite interface becomes smaller, while the other at the perovskite/PC_61_BM interface changes to the opposite direction, that is, pointing towards the perovskite. Hence, the former becomes less favorable whereas the latter becomes more favorable for charge transport. When the unintentional doping type of the perovskite changes from n‐ to (slight) p‐type (Figure S1d,e, Supporting Information), both increasing *E* at the perovskite/PC_61_BM interface and decreasing *E* at the PEDOT:PSS/perovskite interface become more favorable for charge transport. However, when the perovskite is heavily doped with p‐type (until the Fermi level lies below the LUMO of PEDOT:PSS) (Figure S1f, Supporting Information), the interfacial *E* at the PEDOT:PSS/perovskite interface points away from PEDOT:PSS, which is detrimental to charge transport. A similar explanation can be found for the regular structure, and the detailed analysis is included in Note S1, Supporting Information. Moreover, despite the possible large strength of the interfacial *E* (up to 3946 kV cm^−1^), the depletion width, where the interfacial *E* is located, is relatively narrow, as shown in Figure 2. Therefore, heavily doped perovskite is not desirable because the interfacial *E*_s_ are opposite in directions, always causing a negative influence on carrier separation and transportation at one interface.

In addition to the above factors, the unintentional doping of the perovskite is usually accompanied by the undesirable results of a high defect density, which cause non‐radiative recombination in device. Therefore, although heavier doping generally leads to a stronger interfacial *E* at one side and an improved charge separation, in reality, the defect density also increases with the doping density.^[^ ^14^, ^28^ ^]^ Therefore, the heavier doping causes a higher recombination rate, and the positive effect of the interfacial *E* is compromised. From the above simulated results, the device performance is optimized by simultaneously considering various factors, including the direction and strength of the interfacial *E* as well as the defect densities. This also indicates that researchers can optimize the device performance by changing the synthesis process of the perovskite, taking advantage of the interfacial *E* according to the regular or inverted device structures.

**Figure 3:**
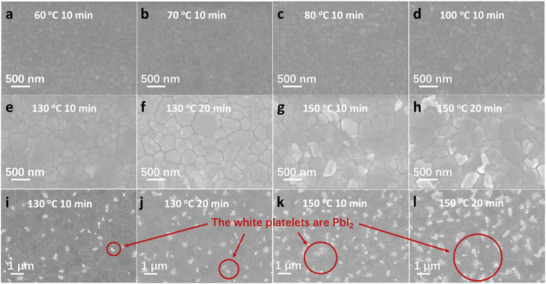
SEM images of MAPbI_3_ films on ITO glass with different annealing times and temperatures of a) 60 °C for 10 min, b) 70 °C for 10 min, c) 80 °C for 10 min, d) 100 °C for 10 min, e) 130 °C for 10 min, f) 130 °C for 20 min, g) 150 °C for 10 min, and h) 150 °C for 20 min. Larger observation view is also provided as shown in i) 130 °C for 10 min, j) 130 °C for 20 min, k) 150 °C for 10 min, and l) 150 °C for 20 min. The scale bars are marked on individual images.

To effectively control the interfacial *E*, we constructed heterojunctions at the interfaces of perovskite and respective transport layers, which are formed by tuning the unintentional doping type and density of the perovskite. The perovskite films were prepared by a one‐step solution‐processed deposition method using PbI_2_ and CH_3_NH_3_I solutions. To realize effective doping control of the perovskite from p‐ to n‐type, the annealing time was changed from 10 to 20 min, and the temperature was varied from 60 to 150 °C. The SEM images in **Figure** **3** show the perovskite thin film morphology. The films exhibit a high‐quality polycrystalline nature with multiple grains compactly covering the substrate without noticeable pinholes. The grain size increased as the annealing time increased from 10 to 20 min, and the temperature increased from 60 to 150 °C. White platelets, which were proven to be PbI_2_ by X‐ray diffraction (XRD) patterns (Figure S3, Supporting Information), were also found in the films fabricated at longer annealing times or higher temperatures. They appeared as the annealing temperature reached 130 °C, and the density of the PbI_2_ increased when the annealing temperature reached 150 °C with an annealing time of 20 min. Similar trends have also been observed in previous studies.^[^ ^14^, ^28^ ^]^

**Figure 4:**
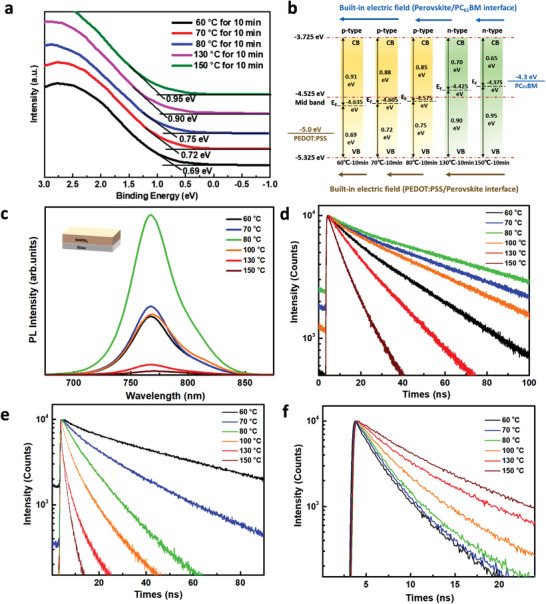
a) XPS of perovskite films fabricated under various annealing conditions. b) Illustration of the strength of the interfacial *E* at PEDOT:PSS/perovskite and perovskite/PC_61_BM interfaces. c) Photoluminescence (PL) and d) time‐resolved photoluminescence (TRPL) measurements of the perovskite under various annealing conditions. e) TRPL measurement of the PEDOT:PSS/perovskite sample and f) perovskite/PC_61_BM sample with perovskite films annealed at various temperatures.

To reveal the doping nature of the perovskite film, X‐ray photoelectron spectroscopy (XPS) measurements were performed on the perovskite films prepared on ITO glass substrates. As shown in **Figure** **4**a,b, the Fermi level of the perovskite goes up (away from the VB) with an increase in the annealing temperature and time. Because the bandgap of the perovskite is 1.6 eV,^[^ ^37^ ^]^ it is clearly observed that the perovskite changes from p‐type doping to intrinsic, and then n‐type doping with increasing annealing temperature or time. As shown in Figure 4b, the Fermi level is located at 0.69 eV away from the VB when the annealing temperature is 60 °C, which reveals p‐type doping. With an increase in the annealing temperature, the Fermi level shifts closer to the CB. For the sample annealed at 80 °C for 10 min, the Fermi level is 0.75 eV above the VB which demonstrates slight p‐type doping. With further increase in the annealing temperature to 150 °C for 10 min, the Fermi level rises to 0.65 eV below the CB, resulting in an n‐type doped perovskite thin film. Moreover, the elemental ratios of iodine and lead are summarized and listed in Table S3, Supporting Information, to support the doping type variation owing to the changes of the annealing time and temperature. The XPS and ultraviolet photoelectron spectroscopy (UPS) valence spectra of the ITO/PEDOT:PSS/perovskite and FTO/TiO_2_/perovskite were also characterized, as shown in Figure S4, Supporting Information, and the different types of transport layers have negligible influence on the energy levels of the perovskite.

The PL and TRPL measurements are employed to investigate the quality of perovskite film under different annealing temperature, as shown in Figure 4c,d. The PL spectrum indicates that the defect density first decreases and then increases with increasing annealing temperature and the perovskite film annealed at 80 °C exhibits the highest PL intensity. This trend coincides with the TRPL spectrum, in which the photogenerated carriers recombine through the dopants or defects. The TRPL decay curves are fitted using a bi‐exponential dependence, where the fast process (*τ*_1_) is caused by bimolecular recombination (short carrier lifetime) at the initial stage, and the slow decay process (*τ*_2_) (long carrier lifetime) is caused by monomolecular recombination at a long‐time scale. In perovskites, bimolecular recombination is caused by the recombination of photogenerated electrons and holes, whereas monomolecular recombination is mainly attributed to trap‐assisted recombination and unintentionally doped carriers. From the decay profiles, both bimolecular and monomolecular recombinations first decrease with annealing temperature and then increase. The bimolecular and monomolecular recombination lifetimes are estimated by fitting the decay curves with the expression *Y* = *A*_0_ + *A*_1_ exp(−*t*/*τ*_1_) + *A*_2_ exp(−*t*/*τ*_2_).^[^ ^38^, ^39^ ^]^ The fitting parameters are listed in **Table** **1**. Both bimolecular and monomolecular recombination rates are high in perovskite films annealed at high temperatures, especially at 150 °C. The samples annealed at 80 °C show the lowest bimolecular and monomolecular recombinations, indicating a low defect density.

**Table 1:** TRPL decay parameters for perovskite/glass samples under different annealing temperatures

Sample	Temperature [°C]	*A*_1_	*τ*_1_ [ns]	*A*_2_	*τ*_2_ [ns]
Glass/MAPbI_3_	60	4167.3	9.5	8412.8	42.1
	70	2755.2	9.3	8911.6	74.4
	80	2567.6	11.3	9167.9	100.5
	100	2579.5	7.8	9297.5	56.7
	130	4799.7	8.8	8625.5	25.3
	150	13 058.9	7.2	3400.2	18.7

To elaborate the influence of interfacial *E* on the carrier extraction and transport, we separately measured the TRPL of the PEDOT:PSS/perovskite and perovskite/PC_61_BM samples, as shown in Figure 4e,f. Due to the change in the Fermi level of the perovskite upon changing the annealing temperature, the strength of interfacial *E* varies, as illustrated in Figure 4b. Regardless of the variation in doping type and density (within our study range), the *E* at the PEDOT:PSS/perovskite interface points from perovskite to PEDOT:PSS, while that at the perovskite/PC_61_BM interface points from PC_61_BM to perovskite, suggesting that the *E*_s_ at both interfaces favor carrier separation/transport. However, the strength of *E* at both interfaces varies with doping type and density, which is confirmed by the TRPL measurements in Figure 4e,f. At the PEDOT:PSS/perovskite interface, the interfacial *E* becomes larger as the perovskite changes from p‐ to n‐type doped conditions (i.e., increasing annealing temperature); hence, faster decay is observed in the TRPL measurement of the PEDOT:PSS/perovskite sample (Figure 4e and **Table** **2**). In contrast, at the perovskite/PC_61_BM interface, the interfacial *E* becomes smaller as the perovskite changes from a p‐ to n‐type doped condition. Hence, a slower decay is observed in the TRPL measurement of the perovskite/PC_61_BM sample (Figure 4f and Table 2).

**Table 2:** TRPL decay parameters for PEDOT:PSS/perovskite and perovskite/PC_61_BM samples under different annealing temperatures

Sample	Temperature [°C]	*A*_1_	*τ*_1_ [ns]	*A*_2_	*τ*_2_ [ns]
Glass/PEDOT:PSS/MAPbI_3_	60	3349.4	8.0	8561.7	56.3
	70	6036.6	6.1	8116.7	26.8
	80	9059.7	5.2	8199.2	13.9
	100	24 806.9	2.8	5318.6	11.2
	130	39 847 400.0	0.4	8858.3	4.4
	150	5 155 190 000.0	0.3	33 662.1	2.0
Glass/MAPbI_3_/PC_61_BM	60	61 503.1	1.6	10 099.4	4.3
	70	47 045.7	2.2	4329.2	5.8
	80	49 354.7	2.1	6222.4	5.5
	100	29 335.3	2.7	5516.6	7.5
	130	19 992.1	3.0	7500.6	9.0
	150	17 295.1	5.6	5710.3	106.8

**Figure 5:**
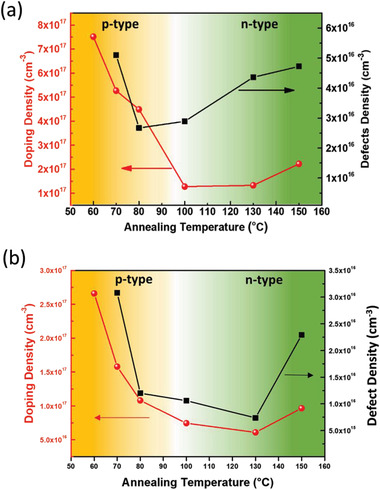
Doping type, density as well as defects density variation of perovskite film as a function of annealing temperature on a) PEDOT:PSS and b) TiO_2_ underlying layer

To estimate the doping density of the perovskite films under various annealing conditions, Mott–Schottky analysis was adopted through the capacitance–voltage measurements on the PEDOT:PSS/perovskite interface, which is widely applied for doping density estimation.^[^ ^30^, ^32^ ^]^ The junction capacitance is calculated from the depletion approximation $\frac{1}{C^{2}}=\frac{2}{\varepsilon \varepsilon_{0}qA^{2}N}\;\left(V_{\mathrm{bi}}-V\right)$, assuming that there are few carriers in the space‐charge region at the junction.^[^ ^40^ ^]^ The *C* is the measured capacitance, *A* (= 0.11 cm^2^) is the active area, *V* is the applied bias, and *ε*_0_ (= 8.8542 × 10^−14^ F cm^−1^) is the free space permittivity, *q* is the elementary charge, and *N* is the doping density. The relative dielectric constant of MAPbI_3_ *ε* (= 32) was obtained from the reference.^[^ ^30^ ^]^ The Mott–Schottky plot $\frac{A^{2}}{C^{2}}=\frac{2}{q\varepsilon \varepsilon_{0}N}\;\left(V_{\mathrm{bi}}-V\right)$ describes a straight line where the intersection on the bias axis determines *V*_bi_ and the slope gives the impurity doping density *N* (Figure S5, Supporting Information). The calculated doping densities at different annealing temperatures are shown in **Figure** **5**a.

The space‐charge limited current (SCLC) measurement was adopted for the defect density calculation. The current density–voltage characteristics of hole‐only devices (ITO/PEDOT:PSS/perovskite/Au) were measured, and the defect density was estimated using the equation *N*_defects_ =  2*εε*_0_ *V*_TFL_/*qL* ^2^,^[^ ^41^, ^42^, ^43^, ^44^, ^45^ ^]^ where *V*_TFL_ is the onset voltage of the trap‐filled limit region, *L* is the perovskite layer thickness, and *q* is the elementary charge (Figure S6, Supporting Information). It was found that the defect density first decreased and then increased with an increase in the annealing temperature, and the lowest defect density was 2.67 × 10^16^ cm^−3^. This defect is principally due to the unintentional doping nature of the perovskite. Regardless of the p‐ or n‐ doping type, the defect density increases with an increase of doping density. Furthermore, the Mott–Schottky plot of TiO_2_/perovskite interface and SCLC of electron‐only devices (FTO/TiO_2_/perovskite/PC_61_BM/Ag) were conducted to analyze the doping density and defect density of perovskite on different underlayers, as shown in Figure 5b and Figures S7 and S8, Supporting Information. The underlayers indeed affect the quality of perovskite film. Compared with PEDOT:PSS underlayer, on average, the defect density of perovskite deposited on TiO_2_ underlayer is slightly lower, and the lowest defect density is obtained at 130 °C annealing temperature, which is corresponding to slightly n‐type condition.

The above analysis proves that the strength of the interfacial *E* can be rationally tuned by unintentional perovskite doping through variations in process parameters such as annealing temperature and time. As a result, the carrier separation/transport at the interfaces of the perovskite and transport layers is enhanced, and the device performance is improved. Based on the findings of the experimental and simulation results, a general design guideline is proposed for both inverted and regular structure perovskite photodiodes, taking advantage of the interfacial *E* to facilitate carrier separation and transport. First, heavily doped perovskite is unfavorable because of the generation of interfacial *E*s in opposite directions at the interfaces and high defect density, leading to a negative influence on the carrier transport. Second, while lightly doped perovskite leads to the same interfacial *E* direction at both HTL/perovskite and perovskite/ETL interfaces, the doping condition that results in a stronger interfacial *E* at the side far from the incident light is more favorable for carrier transport. Third, the defect density should also be considered because unintentional doping of perovskite by process variation (annealing temperature and time in this study) usually results in the formation of defects within the perovskites. The Pb vacancies and I interstitials generate p‐type doping (the atomic ratio of I to Pb exceeds 3, as shown in Table S3, Supporting Information), whereas CH_3_NH_3_ interstitials generate n‐type doping (the atomic ratio of I to Pb is less than 3 as shown in Table S3, Supporting Information). As the annealing temperature and time increased, the Pb vacancy and/or I interstitials decreased, accompanied by the removal of excess CH_3_NH_3_I, resulting in lightly p‐type doped perovskite. With continuously increasing annealing temperature and time, Pb‐richer/I‐poorer conditions occur, and the perovskite film changes from p‐ to n‐type. Therefore, a stronger built‐in electric field results in a higher defect density, and optimal conditions should be reached to balance the positive influence of the interfacial *E* and the negative influence of the defect density.

**Figure 6:**
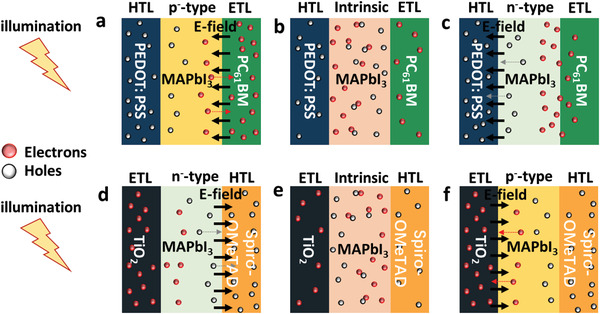
Schematic illustration of the electric field enhanced carrier separation/transport for both inverted from (a) to (c) and regular structures from (d) to (f). The black arrows represent the location and direction of the dominant electric field in different device structures with different doping types.

As shown in **Figure** **6**, for the inverted structure, upon light illumination from the PEDOT:PSS side, the holes can be immediately transported into PEDOT:PSS even without the aid of an interfacial *E*, whereas the electrons should be transported across the entire active layer to reach the ETL side. Along the way, electrons may recombine easily through defects. It is also worth mentioning that despite the interfacial *E* at the PEDOT:PSS/perovskite interface, the depletion region is relatively narrow, and this *E* may not help the transport of electrons to reach the ETL. However, if there is an interfacial *E* near the perovskite/ETL region, the recombination probability can be dramatically reduced, leading to a high device performance. Therefore, a stronger interfacial *E* at the perovskite/ETL side in the inverted structure is highly desirable and a p‐type doped perovskite is favorable, and the corresponding annealing temperature can be rationally selected along with a low defect density. A similar analysis can be applied to a regular structure. With the light coming from the TiO_2_ side, the electrons can immediately be transported to the TiO_2_, and a strong interfacial *E* at the HTL side is highly favorable for hole collection. In this manner, the lightly n‐type doped perovskite will lead to improved device performance.

**Figure 7:**
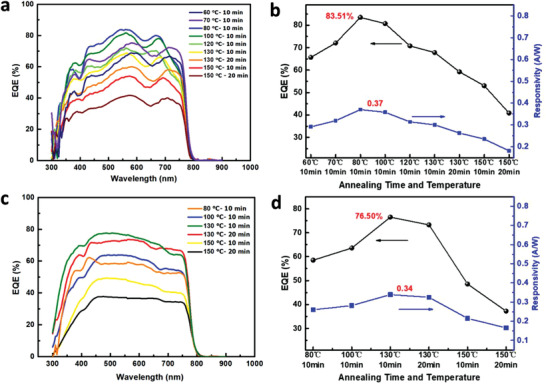
EQEs and responsivities as a function of wavelength for different annealing times and temperatures where (a,b) are for the inverted‐structure photodiode and (c,d) for the regular‐structure photodiode.

Finally, both inverted and regular perovskite photodiodes are fabricated according to the device structures shown in the cross‐sectional views of the devices in Figure 1 to verify our analysis. The device fabrication steps are described in the Materials and Synthesis section. **Figure** **7** presents the external quantum efficiency (EQE) spectra of the fabricated devices under different annealing conditions in the self‐powered mode. In general, reasonable EQEs are achieved for all post‐annealing conditions, which can be attributed to the good perovskite thin film quality, as shown in Figure 3. Optimal performance is achieved when the annealing condition is 80 °C for 10 min for the inverted structure, and the maximum EQE is 83.51% at 550 nm, which is among the highest reported values.^[^ ^46^, ^47^, ^48^, ^49^, ^50^ ^]^ The optimal annealing condition for the regular structure is at the 130 °C for 10 min, where the highest EQE at 550 nm is 76.5%. The responsivity (*R*) is a key parameter for evaluating the performance of the photodiodes, which can be defined as R=EQE/(hc/λ), where h is the Plank constant, *c* is the speed of light in vacuum, and *λ* is the wavelength. As shown in Figure 7, the highest *R* was calculated to be 0.37 and 0.34 A W^−1^ for the inverted and regular structures, respectively. The perovskite doping type, resulting in the highest EQE in the inverted structure, is lightly p‐type, whereas that in the regular structure is lightly n‐type, which corresponds well with the above‐mentioned analysis. From the analysis in Figures 4, 5, 6, it is clear that the built‐in electric fields formed at the active layer/transport layer interface far from the light illumination side is critical for improving the device performance, regardless of device structure. A strong electric field should be formed at the interface far from the illumination side, so that it is favorable for carrier extraction and improved device performance.

**Figure 8:**
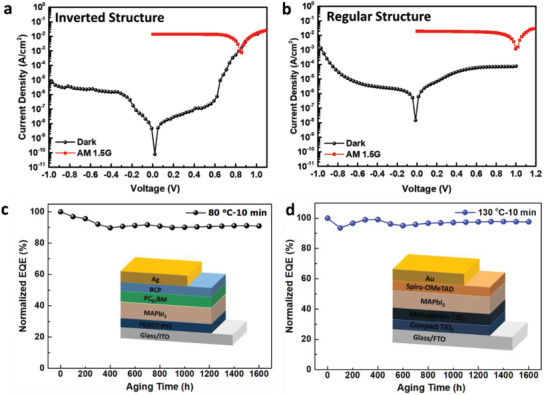
Current density–voltage (*J*–*V*) characterization curves of a) inverted and b) regular photodetectors with corresponding optimal perovskite doping conditions and under AM 1.5G solar illumination with 100 mW cm^−2^. Aging test for the c) inverted and d) regular structure perovskite photodiodes.

**Figures** **8**a and 8b show the dark current and photocurrent densities under illumination of AM 1.5G of inverted and regular perovskite photodiodes with the corresponding optimal doping conditions of perovskite film, under applied voltages ranging from −1 to 1 V. The dark current is usually caused by defects or unwanted impurities located at the surface and grain boundaries. Interestingly, the dark currents in both types of photodiodes are suppressed by the built‐in electric field despite the light perovskite doping. In the self‐powered mode, the lowest dark current can reach 7.95 × 10^−11^ and 1.47 × 10^−8^ A cm^−2^ for inverted‐ and regular‐structure photodiodes, respectively. In addition, the capability to detect weak light was evaluated by specific detectivity (D*). The detectivity can be calculated using the expression D* = R/(2 × *q* × *J*_dark_)^1/2^, where *q* is the elementary charge, *R* is the established responsivity, and *J*_dark_ is the dark current density.^[^ ^51^ ^]^ Due to the small dark current, the inverted and regular perovskite diodes provide maximum values of 7.34 × 10^13^ and 4.96 × 10^12^ Jones, respectively. Under the input of a 532 nm nanosecond pulse laser with a 10 Hz frequency, the inverted and regular photodiodes show rise times of 2 and 40 µs and fall times of 175 µs and 9.9 ms, respectively, for a large active area of 11 mm^2^, as shown in Figure S9, Supporting Information. Herein, the rise time is calculated as the time taken from 10% to 90% of the saturated photocurrent, while the fall time is calculated as the time required for the output signals to decrease from 90% to 10% of their initial values.

Concerning stability, it is still the key issue for polycrystalline perovskite films, ascribing to the defect states in perovskite that induce degradation from moisture, light, and heat. The long‐term stability of both regular and inverted structure perovskite photodiodes is evaluated, as shown in Figure 8. The optimal perovskite synthesis conditions were chosen as 80 and 130 °C for 10 min for the inverted and regular structures, respectively. After fabrication, the EQEs of the respective photodiodes were immediately measured as 83.51% and 76.5%, respectively, which were set as the reference values. The unencapsulated inverted and regular photodiodes performance was evaluated after being stored in ambient air (25 °C and 40 ± 10% relative humidity) for 1600 h. Each new measured EQE was recorded and compared with the initial value. As shown in Figure 8, the EQE evolution over time reveals that both devices can work efficiently over long periods. The results show that the EQE of inverted structure maintained ≈90.92% of the initial EQE after 1600 h, while that of regular structure maintained 97.6% of the initial value. Therefore, both the regular and inverted perovskite photodiodes possess excellent long‐term stability, which can be ascribed to the compactness of the perovskite layer with minimal defect density. Furthermore, the built‐in electric field is favorable for charge separation at interfaces and suppresses ion migration and accumulation, which in turn enhances device stability.

## 3 Conclusion

In this study, the built‐in electric fields formed at the active and transport layer interfaces caused by unintentional doping of the perovskite are systematically investigated, and their influence on photodiode performance is investigated. Both the numerical model and experiments are performed to determine a design guideline for optimized device performance. Favorable charge carrier separation, extraction, and transport are found when a strong built‐in electric field exists at the interface away from the light incidence side. With control of unintentional doping, the interfacial electric fields sweep the carriers to the respective electrodes in favor of carrier transport and collection in both regular‐ and inverted‐structure perovskite photodiodes. By varying the annealing temperature from 60 to 150 °C, the doping type of perovskite changes from p‐ to n‐type, while doping density first decreases and then increases with increased annealing temperature. Due to the nature of the unintentional doping, heavily p‐ or n‐type doped perovskite films are accompanied with a high defect density, which is unsuitable for efficient photodiodes, despite a very strong electric field. Based on the proposed optimal design guideline, efficient inverted and regular perovskite photodiodes are fabricated. The EQEs of 83.51% and 76.5% and the responsivity of 0.37 and 0.34 A W^−1^, being among the highest reported values, are obtained in the inverted‐ and regular‐structure photodiodes, respectively. In the self‐powered mode, the dark current reaches 7.95 × 10^−11^ and 1.47 × 10^−8^ A cm^−2^, providing a high detectivity of 7.34 × 10^13^ and 4.96 × 10^12^ Jones for inverted and regular structures, respectively, along with an excellent long‐term stability of at least 1600 h. Manifesting the origin of the built‐in electric field stems from the unintentional doping of perovskite, this optimization strategy is compatible with existing materials and device architectures, and hence, leads to substantial potential applications in perovskite based optoelectronic devices, in additional to photodiodes.

## 4 Experimental Section

### Preparation of the Perovskite Layer

Perovskite was deposited on the substrate using an anti‐solvent method. From this process, perovskite films with various doping densities were obtained with PbI_2_/MAI ratios of 1.0. The perovskite precursor solution was prepared by dissolving MAI (1.25 mol L^−1^) and PbI_2_ (1.25 mol L^−1^) in a mixed solvent of anhydrous DMF/DMSO volume ratio of 4:1 at room temperature in a N_2_ glovebox. The solution was spin‐coated at 4000 rpm for 20 s. During the spin‐coating step, 120 µL of anisole was poured on the surface 15 s before the end. The pale‐yellow transparent films were converted into brownish‐red perovskite films by annealing at different conditions.

### Preparation of the Inverted Photodiodes (ITO/PEDOT:PSS/MAPbI_3_/PC_61_BM/BCP/Ag)

ITO glass was cleaned in sequence with detergent, deionized water, acetone, and isopropyl alcohol for 15 min, and then dried with N_2_ gas. Before depositing the hole transporting layer, the substrate was treated with UV‐ozone for 15 min. To fabricate the PEDOT:PSS layer, the PEDOT:PSS solution was spin‐coated on ITO glass at a speed of 5000 rpm for 45 s, and then the prepared films were heated at 140 °C for 10 min. Perovskite precursor solution was prepared on ITO/PEDOT:PSS substrate in the inverted photodiodes. To fabricate the PC_61_BM layer, PC_61_BM in anhydrous chlorobenzene solution (20 mg mL^−1^) was spin‐coated on ITO/PEDOT:PSS/MAPbI_3_ at a speed of 1000 rpm for 60 s. To fabricate the BCP layer, BCP in ethanol solution (0.5 mg mL^−1^) was spin‐coated on the PC_61_BM surface at a speed of 4000 rpm for 40 s. Finally, a 100 nm Ag electrode was thermally evaporated on the BCP layer.

### Preparation of the Regular Photodiodes (FTO/Compact TiO_2_/Mesoporous TiO_2_/MAPbI_3_/Spiro‐OMeTAD/Au)

FTO glass was cleaned in the same manner as ITO glass, and before depositing the ETL, the substrate was treated with UV‐ozone for 15 min. The TiO_2_ compact layer was spin‐coated onto the FTO substrate using 0.15 mol L^−1^ titanium diisopropoxidebis (acetylacetonate) in 1‐butanol at 2000 rpm for 20 s, and dried at 120 °C for 10 min. The dried samples were then annealed at 500 °C for 30 min. A mesoporous TiO_2_ layer was deposited onto the compact TiO_2_ layer by spin‐coating a diluted TiO_2_ paste (Dyesol, 30 NR paste) in ethanol (1:9) at 4000 rpm for 20 s and annealed at 500 °C for 30 min. After cooling to 120 °C, the samples were immediately transferred to a N_2_ glovebox for the deposition of the other layers. Perovskite precursor solution was prepared on an FTO/compact TiO_2_/mesoporous TiO_2_ substrate in regular photodiodes. The spiro‐OMeTAD hole‐transport layer solution was prepared by mixing 72.3 mg spiro‐OMeTAD, 28.8 µL 4‐tert‐butylpyridine, and 17.5 µL lithiumbis(trifluoromethanesulfonyl)imide (Li‐TFSI) solution (520 mg Li‐TFSI in 1 mL acetonitrile), and 8.8 µL [tris(2‐(1H‐pyrazol‐1‐yl)‐4‐tert‐butylpyridine)‐cobalt(III) tris‐(bis (trifluoromethylsulfonyl)imide) (FK209) solution (300 mg in 1 mL of acetonitrile) in 1 ml chlorobenzene. The solution was then spin‐coated at 3500 rpm for 20 s. Finally, an 80 nm Au electrode was thermally evaporated on the hole‐transport layer.

### Material Characterization

The surface morphologies of the as‐prepared MAPbI_3_ films and the cross‐sectional morphology of the PDs were characterized by SEM (ZEISS Gemini‐300). The chemical compositions and structures of the perovskite films were analyzed by X‐ray diffraction (Rigaku SmartLab 9 kW system, Cu‐K*α* radiation *λ* = 0.154 nm), and the samples were scanned from 10° < 2*θ* < 40°. The valence and XPS spectra of the perovskite films were measured by XPS (Thermo K‐Alpha+). All spectra were shifted to account for sample charging using inorganic carbon at 284.80 eV as a reference. UPS spectra were measured using He I excitation (21.22 eV) and recorded with a constant pass energy of 3 eV in an ultrahigh vacuum chamber (2 × 10^−8^ mbar) of the XPS instrument (Thermo Fisher, ESCALAB 250Xi). The UPS binding energies were referenced to the Fermi edge of Au. The steady‐state PL spectra of the perovskite films and TRPL decay were measured using a FluoTime 300 under the irradiation of a 532 nm pulse laser. The absorption spectra of the perovskite films were measured using a UV/Vis/NIR spectrophotometer (PerkinElmer Lambda 950).

### Photodiode Characterization

The EQE spectra were recorded in a N_2_ glovebox using an Enli Technology (Taiwan) EQE measurement system (QE‐R) in AC mode under illumination with monochromatic light from a xenon arc lamp, and the light intensity at each wavelength was calibrated with a standard Si photovoltaic cell. The current‐voltage curves in the dark were measured in ambient air using a source meter (Keithley 4200) without irradiation with forward (−1 to 1 V) scans, and the step voltage was fixed at 20 mV. The photo‐response curves of photodiodes under a pulse laser (532 nm) were measured using a mixed domain oscilloscope (Tektronix, MDO3034). The capacitance–voltage curves in the dark were measured using a precision LCR meter 4284A (Hewlett‐Packard, Japan). All the photodiodes had no encapsulation and employed a mask with an aperture area of 0.11 cm^2^ when depositing metal electrode.

## Conflict of Interest

The authors declare no conflict of interest.

## Author Contributions

D.W., W.L., and H.L. contributed equally to this work. D.W. designed the experiments, and wrote the manuscript. H.L. and W.L. performed the main experiments, characterization, and data analysis. K.S. performed the simulation. H.T., C.S., and X.X. helped with the *C*–*V* and measurements. K.W., X.W.S., and A.K.K.K. contributed to the data analysis, discussed the results, and commented on the manuscript. D.W. and A.K.K.K. supervised the project.

## Supporting information

Supporting Information

## Data Availability

Research data are not shared.
