# Supplementary material for: Universal Strategy for Improving Perovskite Photodiode Performance: Interfacial Built‐In Electric Field Manipulated by Unintentional Doping
Source: Adv Sci (Weinh). 2021 Jul 15;8(18):2101729. doi: 10.1002/advs.202101729 (PMC8456202; doi:10.1002/advs.202101729)
Supplement: Supplementary file 1 — Supporting Information [file ADVS-8-2101729-s001.pdf]

© 2021 Wiley-VCH GmbH

ADVANCED SCIENCE
Open Access

Supporting Information

for *Adv. Sci.,* DOI: 10.1002/advs.202101729

Universal Strategy for Improving Perovskite Photodiode Performance: Interfacial Built-in Electric Field Manipulated by Unintentional Doping

Dan Wu, Wenhui Li, Haochen Liu, Xiangtian Xiao, Kanming Shi, Haodong Tang, Chengwei Shan, Kai Wang*, Xiao Wei Sun, and Aung Ko Ko Kyaw**

Supporting Information

Universal Strategy for Improving Perovskite Photodiodes Performance: Interfacial Built-in Electric Field Manipulated by Unintentional Doping

*Dan Wu**, *Wenhui Li, Haochen Liu, Xiangtian Xiao, Kanming Shi, Haodong Tang, Chengwei Shan, Kai Wang**, *Xiao Wei Sun, Aung Ko Ko Kyaw**

Prof. D. Wu, Dr. W. Li, H. Liu, X. Xiao, H. Tang, C. Shan, Prof. K. Wang, Prof. X. W. Sun, Prof. A. K. K. Kyaw
Guangdong University Key Laboratory for Advanced Quantum Dot Displays, Shenzhen Key Laboratory for Advanced Quantum Dot Displays and Lighting, Department of Electrical & Electronic Engineering, Southern University of Science and Technology, Xueyuan Blvd. 1088, Shenzhen, 518055, People's Republic of China
Email: wudan@sztu.edu.cn; wangk@sustech.edu.cn; aung@sustech.edu.cn

Prof. D. Wu
College of New Materials and New Energies, Shenzhen Technology University, Lantian Road 3002, Shenzhen, 518118, People's Republic of China

K. Shi
Light, Nanomaterials, Nanotechnologies (L2n) Laboratory, CNRS ERL 7004, and Department of Optical Nanotechnologies, University of Technology of Troyes, Troyes, 10004, France

D. Wu, W. Li and H. Liu contributed equally to this work.

Table S1. The parameters used for evaluation of the built-in electric fields for inverted structure perovskite diode

	PEDOT: PSS	$MAPbI_3$	$PC_{61}BM$	BCP
Thickness (nm)	10	360	90	5
LUMO (eV)	-3.6	-3.725	-4.3	-3
HOMO (eV)	-5.0	-5.325	-5.3	-6.5
Dielectric constant	3.5	25	3.5	3
N_0 (cm^{-3})	1E21	2.5E18	1E20	1E20
Acceptor doping (cm^{-3})	1E20	/	/	/
Donor doping (cm^{-3})	/	/	1E17	1E19
Electrons mobility (cm^2/Vs)	8E-4	10	1E-3	1E-6
Holes mobility (cm^2/Vs)	8E-4	10	1E-3	1E-6

Table S2. The parameters used for evaluation of the built-in electric fields for regular structure perovskite diode

	Mesoporous TiO_2	Compact TiO_2	$MAPbI_3$	spiro-OMeTAD
Thickness (nm)	40	160	360	160
LUMO (eV)	-4.1	-4.1	-3.725	-2.9
HOMO (eV)	-7.2	-7.2	-5.325	-5.22
Dielectric constant	100	100	25	3
N_0 (cm^{-3})	4.5E20	4.5E20	2.5E18	1E18
Acceptor doping (cm^{-3})	/	/	/	1E19
Donor doping (cm^{-3})	6.2E18	6.2E18	/	/
Electrons mobility (cm^2/Vs)	0.006	0.006	10	0.001
Holes mobility (cm^2/Vs)	0.006	0.006	10	0.001

Note S1. Built-in electric fields for regular structure perovskite photodiode

For regular structure perovskite photodiode, there are two interfacial *E*s located at each side of the active layer. If the perovskite is unintentionally doped with p-type (until whose Fermi level lies below the HOMO of Spiro-OMeTAD) (**Figure S2 f**) , there is a large interfacial *E* at the TiO_2/Perovskite interface pointing from the TiO_2 to the perovskite and another small one at the Perovskite/Spiro-OMeTAD interface which has an opposite direction. The interfacial *E* at TiO_2/Perovskite interface facilitates the transport of carriers to the respective electrodes whereas the other inhibits the transport of the carriers. When the perovskite is (slightly) p-type doped (whose Fermi level lies above the LUMO of Spiro-OMeTAD)(**Figure S2 d** and **e**), the *E* at TiO_2/Perovskite becomes smaller while the other at Perovskite/Spiro-OMeTAD interface becomes opposite in direction, i.e., pointing from the perovskite to Spiro-OMeTAD. Hence, the former becomes less favorable for the charge transport whereas the latter becomes more favorable. This situation continues when the doping type change from p- to (slight) n-type (**Figure S2 b and c)** until the Fermi level of the perovskite becomes the same level as the conduction band of TiO_2. When the perovskite is further doped with n-type (until whose Fermi level lies above the conduction band of TiO_2)(**Figure S2 a**), the direction of interfacial *E* at TiO_2/Perovskite interface becomes opposite (i.e., pointing from perovskite to TiO_2) while the other at Perovskite/Spiro-OMeTAD interface continues to be larger. Therefore, the former becomes less favorable for the charge transport whereas the latter become more favorable.

Table S3. Element ratio of the lead and iodine by X-ray photoelectron spectroscopy (XPS) under different annealing time and temperatures

**Annealing condition**	**Pb**	**I**	**I/Pb ratio**
60 ℃ - 10 min	12.42%	40.42%	3.25
70 ℃ - 10 min	12.37%	40.65%	3.29
80 ℃ - 10 min	12.68%	40.76%	3.21
100 ℃ - 10 min	14.64%	45.31%	3.10
130 ℃ - 10 min	12.65%	40.42%	2.99
150 ℃ - 20 min	19.27%	55.60%	2.89

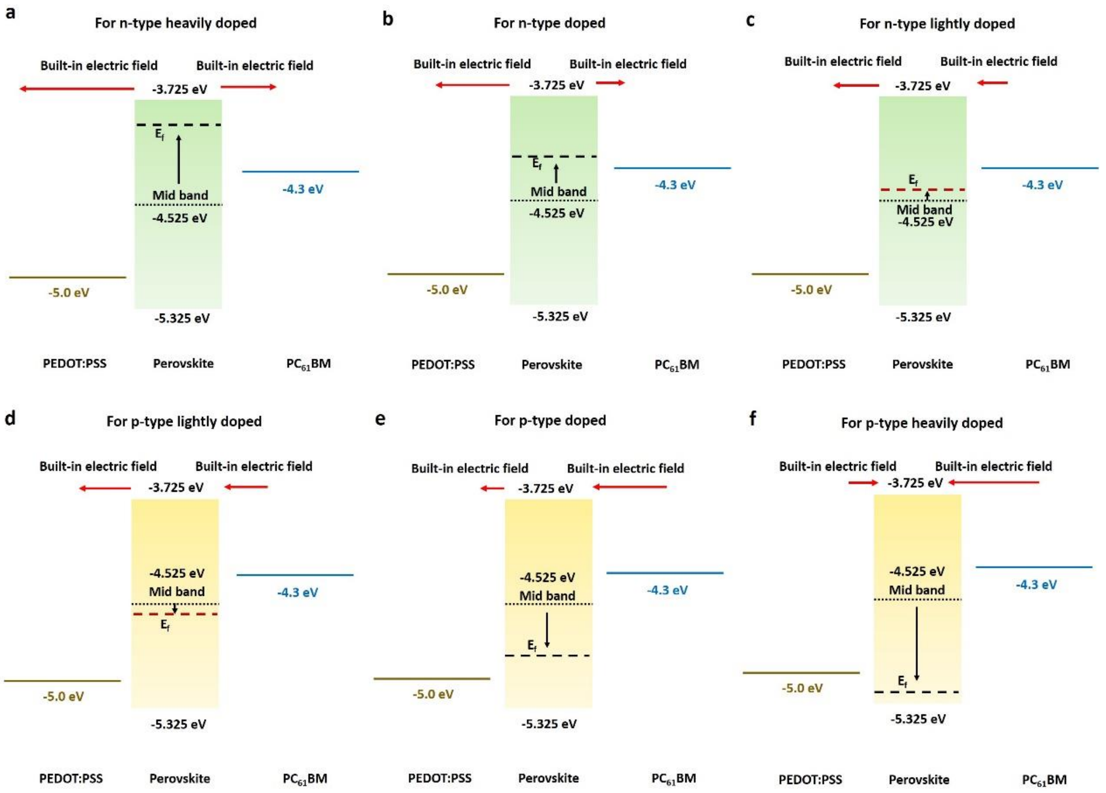

Figure S1. Schematic of the interfacial *E* for inverted-structure perovskite photodiode.

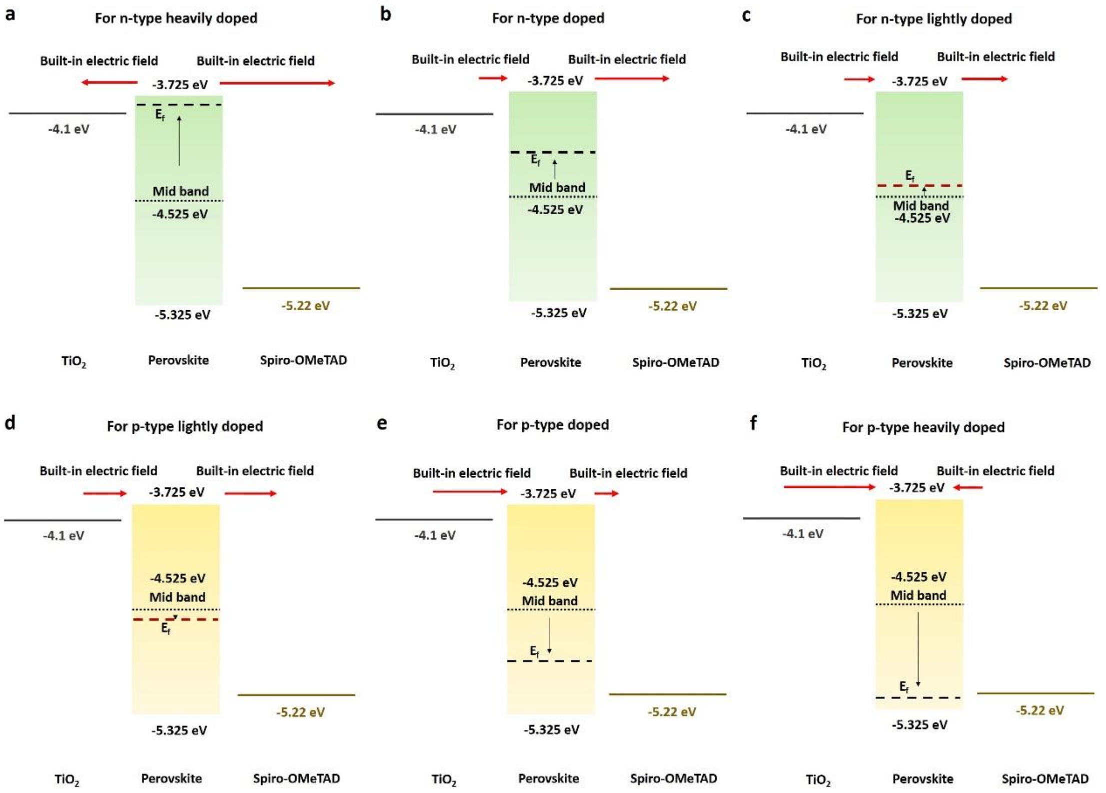

Figure S2. Schematic of the interfacial *E* for regular-structure perovskite photodiode.

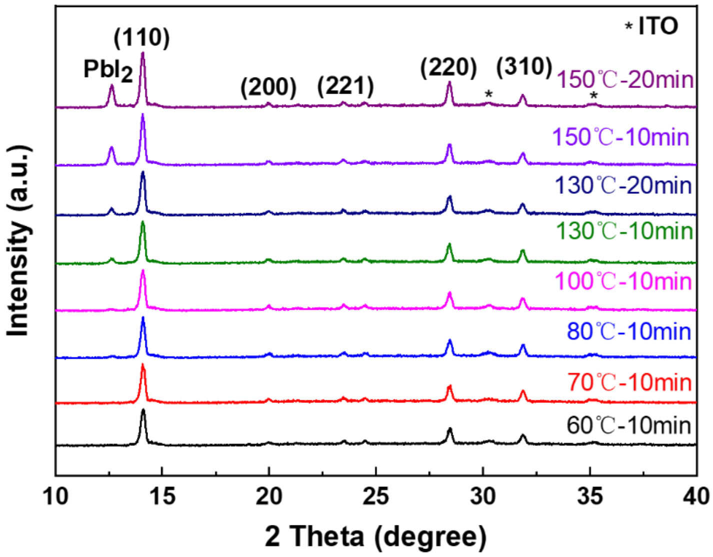

Figure S3. X-ray diffraction (XRD) results of perovskites samples with various annealing time and temperatures.

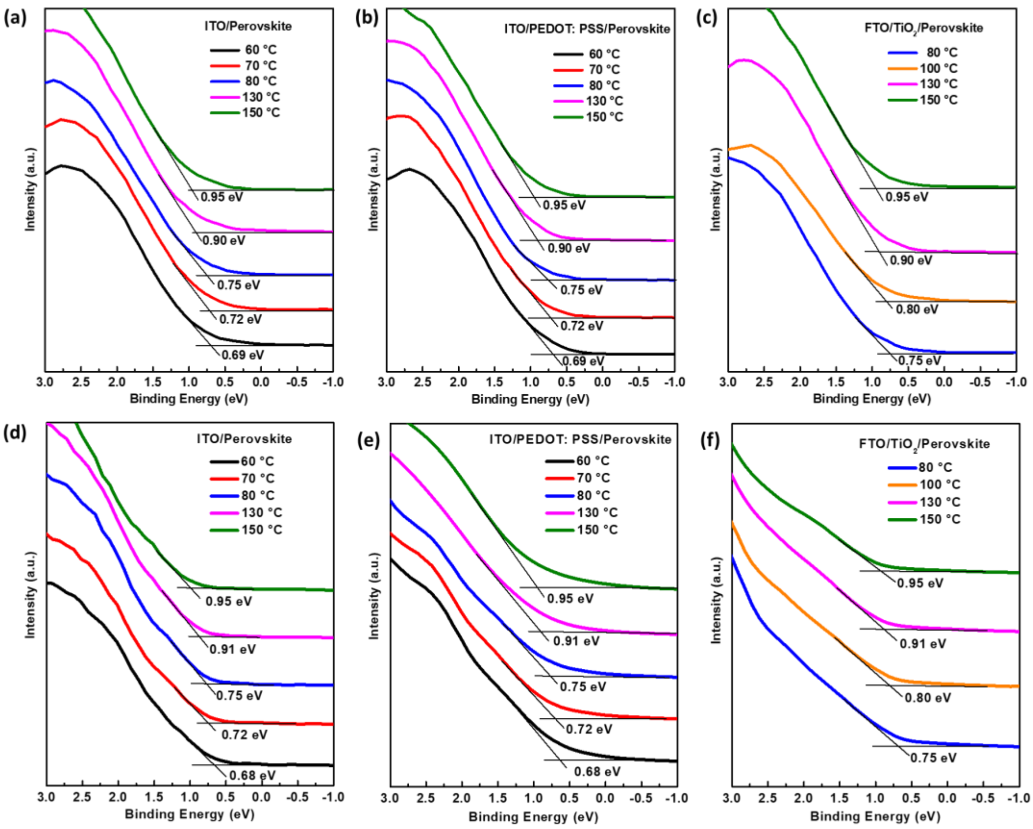

Figure S4. (a-c) X-ray photoelectron spectroscopy (XPS) and (d-f) Ultraviolet photoelectron spectroscopy (UPS) valence spectra of the ITO/Perovskite, ITO/PEDOT:PSS/Perovskite, and FTO/TiO_2/Perovskite samples.

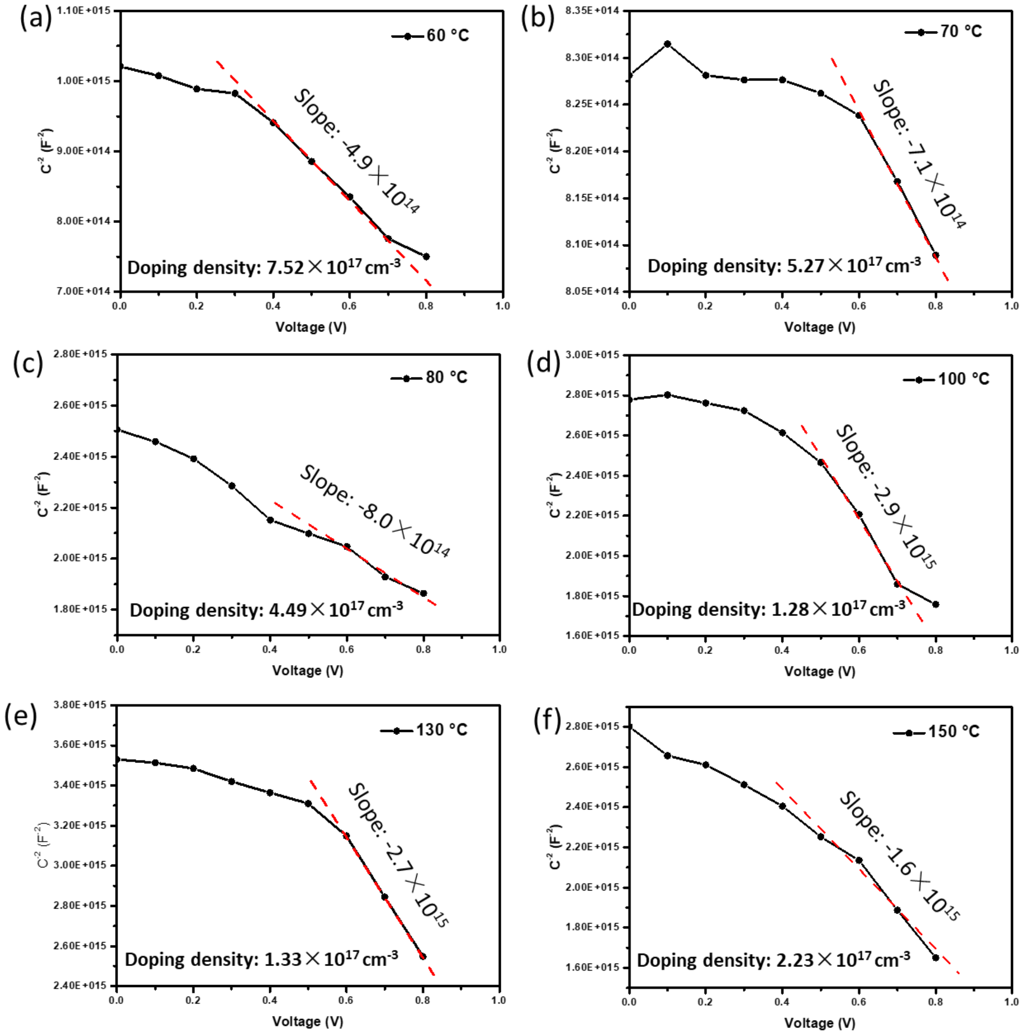

Figure S5. Mott-Schottky plot of control and perovskite films under various annealing conditions measured on PEDOT: PSS/Perovskite interface.

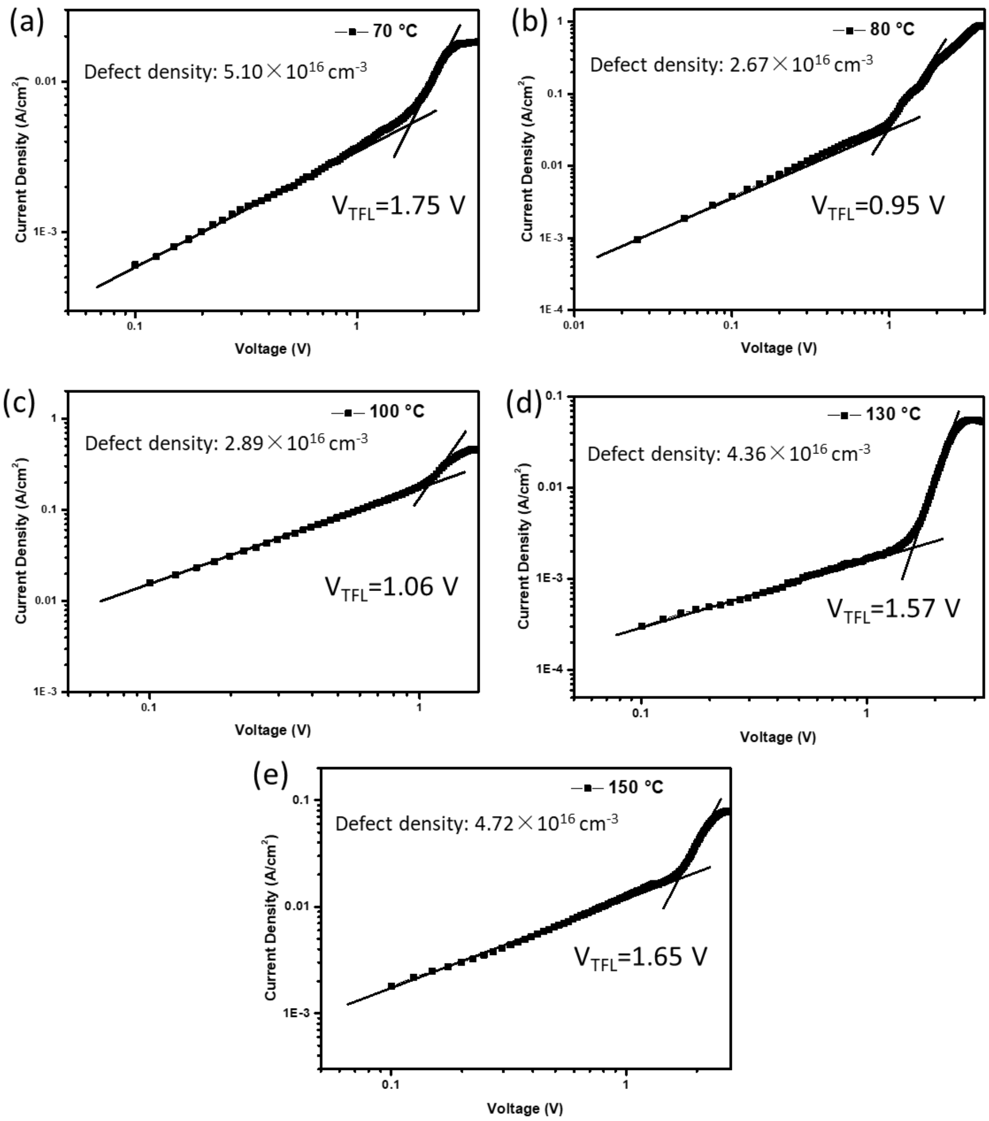

Figure S6. Space-charge limit current for the hole-only devices of control and perovskite under various annealing conditions.

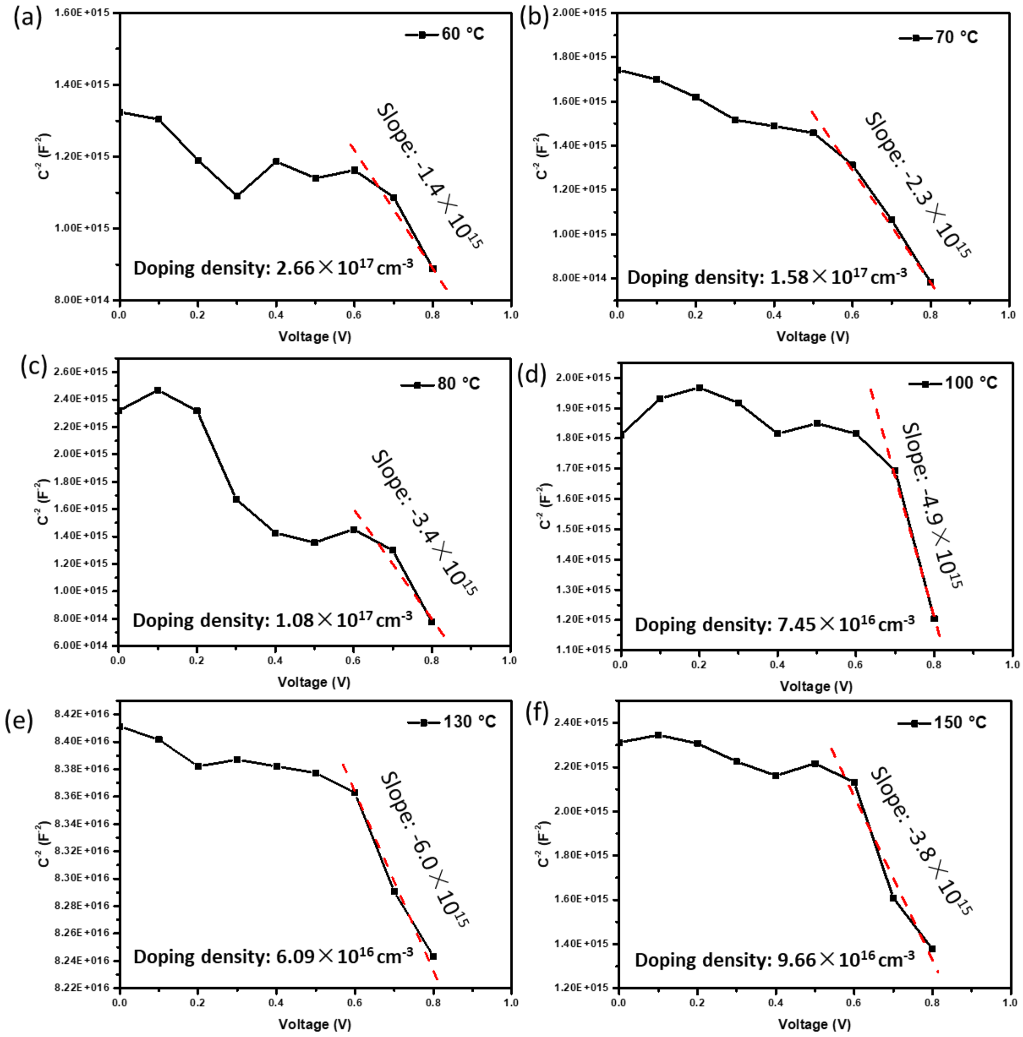

Figure S7. Mott-Schottky plot of control and perovskite films under various annealing conditions measured on TiO_2/Perovskite interface.

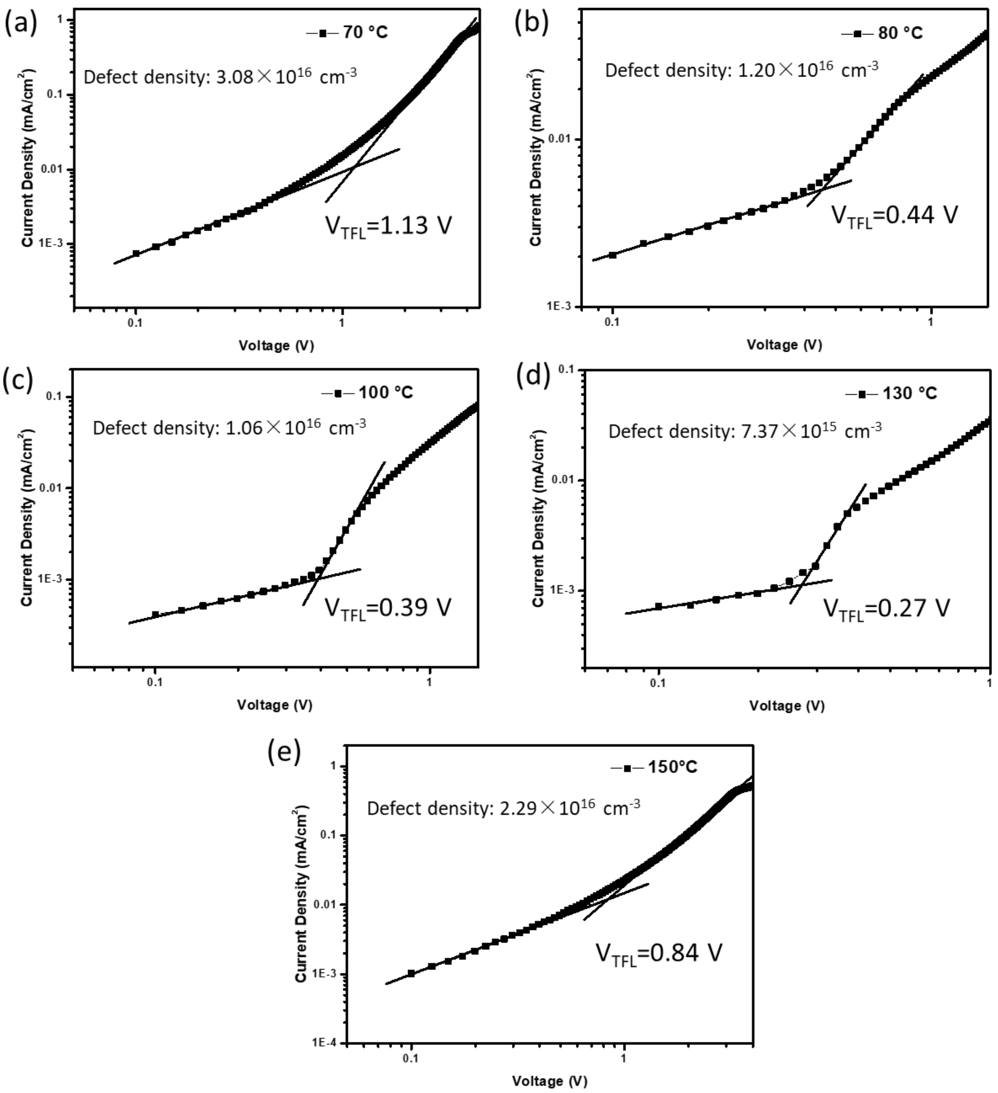

Figure S8. Space-charge limit current for the electron-only devices of control and perovskite under various annealing conditions.

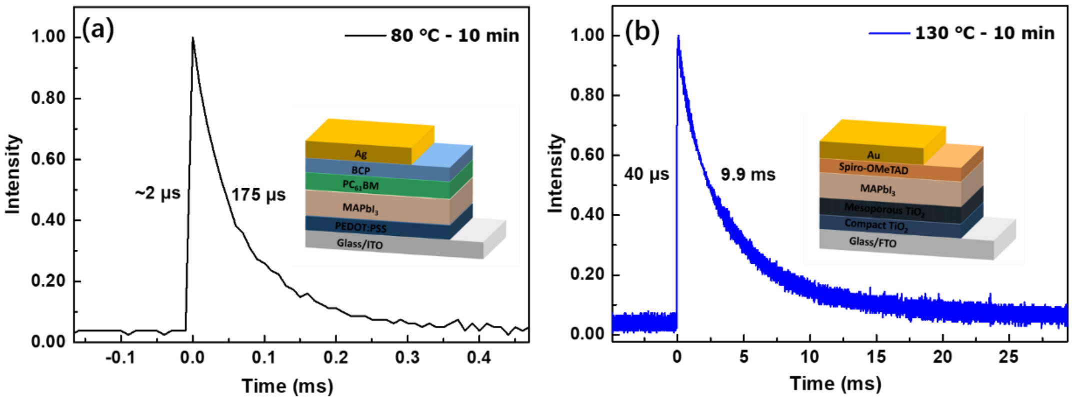

Figure S9. Response time for a) inverted and b) regular structure perovskite photodiodes.
